# State Examination for Medical License

**Published:** 1895-03

**Authors:** 


BUFFALO MEDICAL AND SURGICAL JOURNAL

A MONTHLY REVIEW OF MEDICINE AND SURGERY.

*EDITORS:*

THOMAS LOTHROP, M. D. - - WM. WARREN POTTER, M. D.

All communications, whether of a literary or business nature, should be addressed to the managing editor: 284 FRANKLIN STREET, BUFFALO, N. Y.

VOL. XXXIV. MARCH, 1895. No. 8.

### STATE EXAMINATION FOR MEDICAL LICENSE.

THE annual report of the state examining and licensing board, published in another column, furnishes interesting reading for all physicians who favor improved methods in medical education. This ought to mean every graduated physician, but, sadly enough, it does not if we are to accept their actions and statements, implied or declared, as truthful. That any physician of education should be opposed to state examination for license in this day and age is beyond our understanding.

The report in question embraces the work of the board of examiners for the year ending December 31, 1894, and should not be confounded with the annual report of the regents, which is for the academic year ending July 31, 1894. The difference in time here indicated will account for any apparent discrepancy in annual statistics between the two reports.

The board very generously invites criticism of its work with a view to improve its practices, hence we feel at liberty to make suggestions. One of the most important subjects relating to this examination at the present time is that of the selection of questions to be propounded to the candidates. While in the main the questions submitted by the board have been satisfactory, yet in certain instances it cannot be denied that they have been defective in meaning or faulty in expression. One of the principles to be borne in mind in framing these questions is, or should be, that they are not to be propounded to undergraduates; but, on the other hand, the questions are to partake of the nature and character of post-graduate interrogatories. Moreover, they are not to be framed on a basis of the teachings of the past, nor yet should they be so advanced as to deal with vague theories. They should be eminently practical; they should deal with the medicine of the present and each one should be comprehensive enough to test the applicant's knowledge on the subject dealt with. No question should be asked that permits of a categorical answer, or an answer of such simplicity that a single phrase of two or three words inevitably merits a marking of ten points. Such questions tend to make the candidate's average unduly high.

Each series of questions should be direct and positive rather than indirect and negative. Each one should bring out some prominent principle of the branch of medicine to which it pertains. They should not, however, be too general in their range. Recent graduates in medicine, as a rule, have but little practical knowledge, and their generalizations are based upon theories taught them in the schools; hence, any vagueness in the construction of a question places candidates at an unfair disadvantage.

From all points of view it seems to us that the proper framing and propounding of questions is one of the most important functions with which the board at present has to deal. A great responsibility devolves upon the question committee and especially upon the editor to whom all accepted questions are referred. He must not only prevent the asking of improper questions, but he must be sure that every question is professionally and grammatically correct. This officer has a delicate and important work in hand which cannot be performed in a superficial or hasty manner.

Intimately associated with the subject of appropriate questions is that of proper markings. It matters not how befittingly a group of questions may be propounded if they are inadequately or unbecomingly marked. To place a high value on a mediocre answer is to do the state an injustice, while equal wrong may be done the candidate in undervaluing his efforts. Nevertheless, the aim of the board should be, in our view, constantly toward improved methods and a higher training of physicians; hence, the lines must be drawn tighter and tighter as time advances.

The state commission, confessedly, should not be awarded to imperfectly educated or inadequately equipped physicians, nor should it be withheld from those who have properly earned it. Those cases that lurk on the border line are of the greatest responsibility, and are those wherein the stamina and judgment of the board are brought to their highest test. Doubtful cases can wait. If a candidate is rejected through error, this is of speedy correction and swift remedy, but if the state license is given to one inadequately equipped, the error is irremediable and its results will appear in the annual mortuary statistics of the state. The state has no need of incompetent medical men, and the yearly death-rate will be in direct ratio to their incompetency.

It must be humiliating for the members of the examining board to affix their signatures to a license issued to a man with a defective English education, no matter how readily he may answer the medical questions. Nevertheless, this is one of the defects of our system. The law does not compel a sufficiently high standard of preliminary education to prevent this absurdity, and the colleges do not seem to take sufficient pride in the matter to enforce a high standard of preliminary training in the absence of a law on the subject. Hence, it is with satisfaction that we point to the resolution adopted by the board and appended to its report. It must not be forgotten, however, that the medical practice law has conferred one benefit upon the public that is beyond estimated value. It compelled the colleges to teach three years, instead of two as heretofore; it compelled them to increase the length of the scholastic year, and it enforced an increase in the number of teaching chairs in the schools. These improvements alone are worth more to the people than all the time and money expended during the ten years' struggle for the establishment of the system.

But the end is not yet. The trend of public opinion is in the direction of further lengthened terms for medical study. The Yale Medical School faculty has appointed a committee to prepare a plan for the extension of the present three years' course to four years. Other schools have already adopted the plan, and it will not be long before New York will be taking her place in the procession that is keeping step to the tune of four years of college training. Let our colleges put on their marching boots and be ready to head the column.

But we digress. We have nothing but commendation to bestow upon the board for its work during the three years and a half of its existence. It has overcome difficulties that seemed well-nigh impassable and it has achieved success in spite of opposition. Let it go on with its good work, let it ever increase its efficiency, elevate its standards and improve the status of the profession in the Empire State. It will then deserve to receive the approbation of every physician worthy of the name within her borders.

